# Accountability for women’s, children’s and adolescents’ health in the Sustainable Development Goal era

**DOI:** 10.1186/s12889-016-3399-9

**Published:** 2016-09-12

**Authors:** Carmen Barroso, Winfred Lichuma, Elizabeth Mason, Pali Lehohla, Vinod K. Paul, Giorgi Pkhakadze, Dakshitha Wickremarathne, Alicia Eli Yamin

**Affiliations:** United Nations Secretary-General’s Independent Accountability Panel (IAP) for the Global Strategy for Women’s, Children’s and Adolescents’ Health, IAP Secretariat, The Partnership for Maternal, Newborn and Child Health, Avenue Appia, 1211 Geneva, Switzerland

## Background

Commitment to the Millennium Development Goals (MDGs) in 2000 resulted in political momentum, new investments and mobilisation to implement practical steps needed to meet the framework of eight MDG goals. MDG 5 (improve maternal health) and MDG 4 (reduce child mortality) focused on improving women’s and children’s health, with newborn health not added until the mid-2000s. The MDG era saw vast improvements in maternal and child survival, with global under-five mortality rates declining by more than half, from 90 to 43 deaths per 1000 live births between 1990 and 2015, and a 45 % decline in maternal mortality ratio worldwide, with more reductions occurring since 2000 [1]. However, as the MDGs draw to a close, the annual death toll, most of which could have been prevented, remains unacceptably high with 303,000 maternal deaths, 2.6 million stillbirths, 5.9 million deaths in children under the age of five - including 2.7 million newborn death - and 1.3 million adolescent deaths [2–7]. It also remains very unequally distributed, with poor countries and poor individuals in middle income countries shouldering the largest burden.

**Fig. 1:**
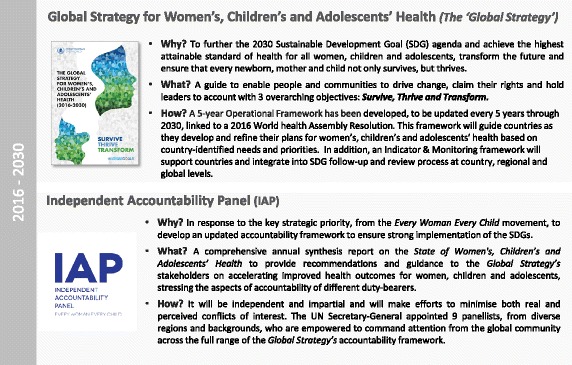
Overview of the Global Strategy for Women’s, Children’s and Adolescents’ Health, and the Independent Accountability Panel

The Sustainable Development Goals (SDGs) launched in September 2015, have received commitment from all governments to implement an ambitious agenda of 17 goals over the next 15 years, with SDG 3 focused on ensuring healthy lives and promoting well-being for all. SDG 3 retains a focus on survival, but also sets goals to reduce disability and illnesses that hinder people from reaching their full potential, resulting in enormous loss and costs for countries, and importantly includes an equity parameter with universal health coverage and leave no-one behind. The new Global Strategy for Women’s, Children’s and Adolescents’ health (Global Strategy) and its operational framework are aligned with the SDGs, and provide an evidence-based roadmap for ending preventable deaths of women, children and adolescents by 2030 (Fig. 1) [8]. The three pillars of the Global Strategy – survive, thrive and transform - aim to go beyond ending preventable mortality, to ensure that all women, children and adolescents’ health and well-being are transformed to shape a more prosperous and sustainable future.

Evidence from this journal supplement shows that further global and national level accountability is needed for tackling persisting inequalities and uneven progress for women’s and children’s health in the post-2015 era [9]. To achieve optimal national level accountability there is an urgent need for improvements in the quantity and quality of subnational data, as demonstrated in this supplement by Singh et al. and Armstrong et al. in their analyses of change in health systems inputs and coverage of interventions for women’s and children’s health in Tanzania [10, 11], and by Huicho et al. in their district-level analysis of reductions in neonatal mortality rates in Peru care showing remaining gaps for urban and rural populations [12]. It is imperative to address data gaps, as well to implement high-impact interventions with an increased focus on equity and quality to ensure that women, children and adolescents are not left behind in the SDG era and that stillbirths are counted.

In order to achieve accountability for women’s, children’s and adolescents’ health, it is key to focus on political attention and leadership, promotion of individual and community voices, investment, implementation at scale, and evaluation [13]. Also key is for nations and stakeholders to commit to intermediate milestones, key to measuring progress. To drive this accountability agenda, the Global Strategy interlinks global and country level accountability under a Unified Accountability Framework for resources, results and rights at the country, regional and global levels and between different stakeholders and sectors. The World Health Organisation with Every Woman Every Child, H6 and other partners have developed a “Global Strategy Indicator and Monitoring Framework”[14], aligned with SDG indicators and established global initiatives to minimise reporting burden, which will be implemented in part via the Independent Accountability Panel (IAP). The IAP is comprised of nine diverse panellists empowered to command global attention to the issues comprising the Global Strategy’s survive, transform and thrive themes, and will work to monitor, review, remedy and action progress towards women’s, children’s and adolescents’ health [9].

In an effort to harmonise global reporting and minimise the reporting burden on countries, the IAP will prepare a comprehensive annual synthesis report on the State of Women’s Children’s and Adolescents’ Health, using information routinely provided from United Nations agencies, independent monitoring groups and bodies such as National Human Rights Institutions (Fig. 1). This annual report will be developed independently and transparently to provide the global community with the best available evidence on progress towards achieving the Global Strategy’s objectives, together with the relevant SDGs, including SDG 16 to “develop effective, accountable and transparent institutions” [8]. The report will provide recommendations and guidance to the Global Strategy’s stakeholders on accelerating improved health outcomes for women, children and adolescents, stressing the aspects of accountability of different duty-bearers in doing so. As part of the Global Strategy’s accountability framework, the IAP is charged with ensuring the global and national-level communities monitor, review and act to keep women’s, children’s and adolescent’s health at the heart of the SDG agenda, and to ensure they go beyond survival to thriving and transforming in the post-2015 era. Better data, better use of data and national leadership are crucial to true accountability.
